# Paired kidney donation: are we going beyond reasonable limits in living-donor transplantation?

**DOI:** 10.1590/2175-8239-JBN-2021-0230

**Published:** 2022-01-19

**Authors:** José Medina-Pestana, Mario Abbud-Filho, Valter Duro Garcia, Renato Demarchi Foresto, Lúcio R. Requião-Moura

**Affiliations:** 1 Fundação Oswaldo Ramos, Hospital do Rim, São Paulo, SP, Brasil.; 2 Universidade Federal de São Paulo, Escola Paulista de Medicina, Disciplina de Nefrologia, São Paulo, SP, Brasil.; 3 Fundação Faculdade Regional de Medicina de São José do Rio Preto, Faculdade de Medicina, Centro de Transplante de Órgãos e Tecidos, Hospital de Base, São José do Rio Preto, SP, Brasil.; 4 Centro de Transplante Renal, Santa Casa de Porto Alegre, Porto Alegre, RS, Brasil.

**Keywords:** Kidney Transplantation, Paired Kidney Donation, Living Donors, Transplante de Rim, Doação Renal Pareada, Doadores Vivos

## Abstract

The growing demand for transplant kidneys requires strategies to increase organ supply and avoid long waiting periods on the list. The increase in the number of transplants from living donors involves the growth in the use of unrelated donors and paired kidney donation. Most of these transplants are performed in the USA, where they already represent, respectively, 34% and 16% of total transplants from living donors. In Latin America, and especially in Brazil, there is no collective enthusiasm for these modalities, either at the request of transplanters or that of the community, with the region's priority being to increase transplants from deceased donors, which growth can be up to three-fold. Concerning transplants from matched donors, the possible conflicting results between donors can generate public challenges and they risk compromise the concepts of equal opportunities for transplant candidates, with the possibility of generating resistance to organ donation, especially in regions with socioeconomic limitations and disparities in access to qualified health care and education. This donation model involves challenging ethical and logistical issues, which are subject to questionings, starting with an act of exchange between two pairs until reaching embarrassing proposals, which can compromise the altruistic character of organ donation, and thus not be universally incorporated.

## Resumo

A demanda crescente por rins para transplante requer estratégias para aumentar a oferta de órgãos e evitar longos períodos de espera em lista. O aumento no número de transplantes com doador vivo envolve o crescimento da utilização de doadores não aparentados e a doação renal pareada. A maior parte desses transplantes são realizados nos EUA, onde já representam, respectivamente, 34% e 16% do total de transplantes com doador vivo. Na América Latina, e especialmente no Brasil, não existe entusiasmo coletivo por essas modalidades, quer por demanda dos transplantadores ou da comunidade, sendo prioridade da região incrementar o transplante com doador falecido, cujo crescimento pode ser de até três vezes. Na modalidade de transplantes com doadores pareados, os possíveis resultados conflitantes entre doadores podem gerar questionamentos públicos e riscos que comprometem os conceitos de equidade de oportunidades para os candidatos a transplante, com possibilidade de gerar resistência à doação de órgãos, especialmente em regiões com limitações socioeconômicas e disparidades de acesso aos atendimentos de saúde e educação qualificados. Esse modelo de doação envolve questões éticas e logísticas desafiadoras, que estão sujeitas a questionamentos, começando por um ato de troca entre dois pares até alcançar propostas constrangedoras, o que pode comprometer o caráter altruístico da doação de órgãos, e assim não ser universalmente incorporado.

## Introduction

In certain countries, such as South Korea, the USA, Switzerland, the Netherlands, Australia, Canada and India, donation models have been developed through the paired exchange of donors between two or more pairs to enable transplantation to recipients whose living donors are ABO -incompatible or have positive HLA crossmatch^1^^-^ 6. The approval of this procedure is not universal and, among others, in Japan, paired donation is not allowed, for ethical reasons within that culture^7^. In Brazil, a country with great socioeconomic disparities, there is an additional concern with the repercussions of these models, concerning the stability of a growing national transplant program from deceased donors^8^.

Although controversial, the growth of this model has been based on two pillars: the option for carrying out a transplant from a deceased donor is remote, due to the progressive growth of the waiting list; and both life quality and expectancy provided by transplantation are much better than what is provided by dialysis^9^.

This modality of transplantation, which started more than 20 years ago, was first performed with the exchange between two pairs in the same center in South Korea, in 1991^10^, then followed by the chain of exchanges between several pairs, also in the same center, followed by geographic expansion, until reaching international status, using the same criteria as local exchanges^11^^-^ 16.

The level of criteria flexibilization expanded to include a new feature, the altruistic donor, who, without a chronic renal partner, triggers a sequence of domino transplants by donating to a recipient whose incompatible donor donates to the next compatible recipient, all the way to the end of the chain, when a last donor undergoes nephrectomy for the first on the waiting list for deceased donors^17^.

**Figure 1:**
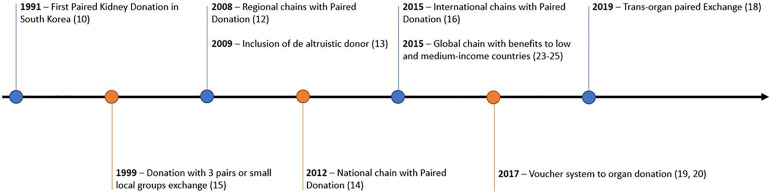
Modalities assimilated in paired donation between 2 pairs in 1991 to the last two concepts involving the global chain of paired donation in 2015 and the paired trans-organ exchange in 2019.

Other bolder modalities, due to the greater clinical and ethical risk, were idealized. In the trans-organ exchange, a potential kidney donor, discarded due to a clinical impediment, but without clinical limitations for donating part of the liver, realizes the paired donation to a recipient whose donor, unable to donate the liver, would be a kidney donor^18^. A little more complex is the anticipated donation, when a donor undergoes nephrectomy to ensure a future donor to his family member with chronic kidney disease, but not yet requiring a transplant, as in the case of a father with a young child with polycystic kidney, which anticipated his donation, guaranteeing a "voucher" for a preemptive transplant to the child when transplantation is needed because of the polycystic disease, decades later^19^^,^ 20. Extremely controversial is the so-called global exchange of kidneys, which proposes the involvement of a pair from a developed country and another from a developing country, with financial limitations that prevent their access to transplantation and specialized monitoring. Savings from suspending the dialysis in the developed country would be directed towards the expenses with carrying out monitoring the transplant for a certain period for the couple from the developing country^21^^-^ 25. Figure 1 depicts the temporal sequence of implementing these exchange proposals between donors.

In addition to the contradictory issues associated with ethics and logistics, there are arguments concerning the risks for the live donor, both immediate and long-term, which, as they are not identical between the exchanged pairs, can result in asymmetric losses and generate conflicts that compromise the concepts of living donation between family members or even compromise the development of transplant programs from deceased donors, especially in countries with more socioeconomic limitations^26^^-^ 28.

## Discussion

The growing demand for kidneys for transplantation and the continuing shortage of donors demand the search for strategies to increase the supply of organs and avoid long waiting periods on the list. In recent years, deceased donor strategies have had greater international success in increasing the pool of donors, particularly using older and expanded criteria donors. Some countries have also increased their pool of deceased donors based on post-cardiac arrest diagnosis, which is limited to countries with better health and logistics programs^29^.

Living donor-based increases involve the growing use of unrelated donors and paired kidney donation, which has been a tool used to overcome immunological incompatibility in the living donor context, with a particular focus on recipients with high lymphocyte panel reactivity, with difficulty finding an HLA compatible donor^30^.

**Figure 2:**
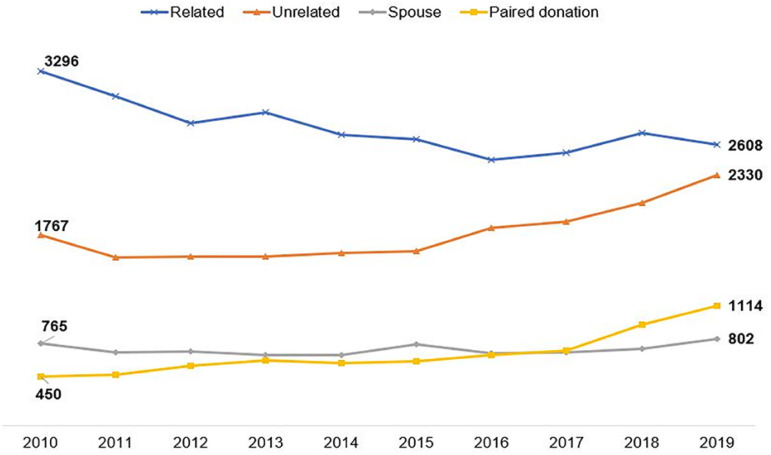
Evolution curves in the number of kidney transplants from living donors performed in the USA between 2010 and 2019, according to the relationship between pairs.

**Figure 3:**
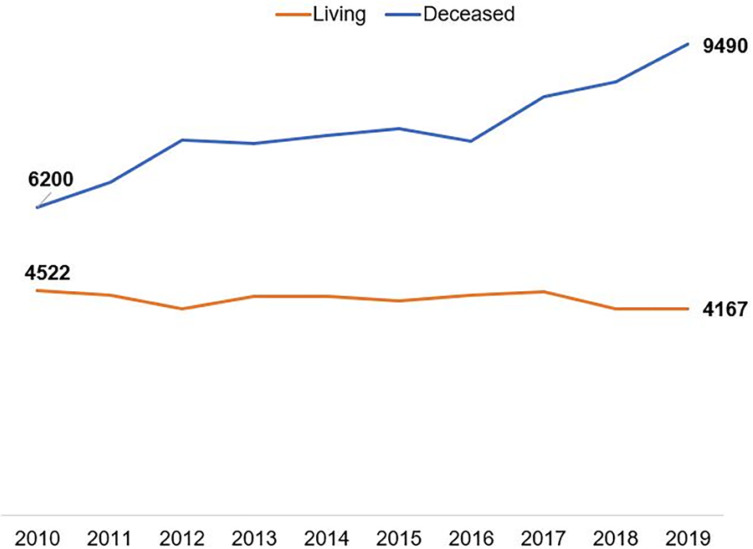
Evolution curves in the number of kidney transplants from living or deceased donors in Latin America, from 2010 to 2019.

**Figure 4:**
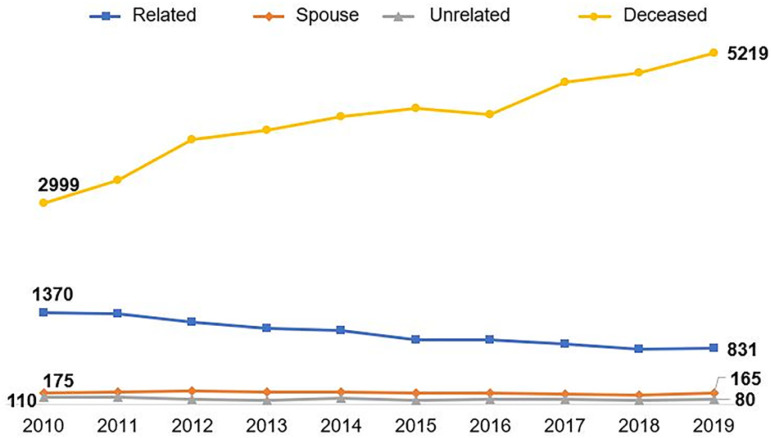
Evolution curves of the number of kidney transplants from living and deceased donors in Brazil, according to the type of donor and the relationship between the pairs.

International data on the number of transplants using the different modalities of paired donation are shown in Figures 2, 3 and 4. The largest number is carried out in the USA, where it is growing and more than a thousand transplants with paired donors are carried out annually, already representing 16% of all transplants from living donors. Figure 2 also shows the growth in the number of transplants with deceased donors in parallel with the number of unrelated donors and the progressive reduction in the number of family donors. The interpretation of the relationship between these numbers over the years, shown in the figure, may suggest a lower willingness to donate among family members, considering the benefits of the other options.

In Latin America, there is no collective enthusiasm, either due to transplantation or community demand for this modality; however, although regulated in Peru, Argentina and Chile, no consistent program is active in the region. On the contrary, there is an increase in the number of transplants from deceased donors (53%) and a reduction in transplants from living donors (7.8%) in the last decade, clearly represented in Figure 3. This trend is even more pronounced in Brazil, where the reduction in the number of living donors was of 35.0%, against a 74.0% increase in transplants from deceased donors in the same period (Figure 4). In addition to the greater focus on the growth of organ harvesting systems from deceased donors, there has been a gradual trend towards a reduction in the number of procedures from living donors, especially unrelated donors, or even young donors, such as children.

Medical procedures are not always safe and without risk, but they must always be based on prudence, considering the individual and the collective benefits and risks they may bring. The proposal of a transplant program with donor exchanges can compromise the development of transplant programs across countries and cultures in different ways^31^. In the US and some European countries with a more effective social care and assistance network and well-established transplant programs from deceased donors, it might be individually challenged, but subject to less risk of interfering with existing programs, such as increasing the transplantation from deceased donor. On the other hand, in countries with different cultures, such as Japan, or with greater social and economic disparity, such as in Latin America, possible conflicting results can generate public challenges that can compromise the concepts of equality of opportunity for transplant candidates and generate resistance to organ donation.

Even nephrectomy for donation is not a risk-free procedure for the living donor, and there is some controversy considering the surgical risk, which mortality is reported to be between 1 in 3 in 10,000 donors, as well as the long-term damage, considering that the current life expectancy for healthy people in a compatible age group for donation is close to 90 years, bringing risks and discomfort to the donor^28^. Many consider that these risks, when taken on by the donor, are based on a consistent and lasting affective relationship, such as the donation that occurs from parents to children.

Concerning these decisions, we must not discard the fact that quality of life, as well as life expectancy in dialysis, especially in the daily modality, compared to transplantation, has been declining^32^, and a transplant from a living donor may not be the best option in many a case. We must also consider that the very successful kidney transplant recipient will still be subjected to permanent immunosuppression, with a high risk of opportunistic and neoplastic diseases, which became very evident due to the lack of vaccine response to covid-19 and the ten-fold higher lethality in this population.

## Conclusion

This transplant modality should be considered, especially in regions with socioeconomic limitations and great disparities in terms of access to quality health care and education. This donation model involves challenging ethical and logistical issues that are subject to further questioning, and which involves, among other factors, the impossibility of guaranteeing both the recipient and the donor the same benefits, as well as the risks between the various exchanged pairs. It started as an act of exchange between two pairs until reaching embarrassing proposals that could compromise the altruistic nature of organ donation.

We must use our judgment ​​and prudence according to our stage of social evolution and not run the risk of jeopardizing the achievement of our society in this highly complex and delicately balanced field of medical practice, because it involves emotional and technical issues that are difficult to understand, such as the diagnosis of brain death, the use of drugs with high risk of health impairment -such as immunosuppressants, and also having dialysis as an option, which, although it provides a life with some limitations, mainly related to the time spent for the procedure almost daily, it may not justify going beyond the limits of safety to benefit only a few^33^.
